# Supplementary material for: STAT3 Inhibits CD103+ cDC1 Vaccine Efficacy in Murine Breast Cancer
Source: Cancers (Basel). 2020 Jan 4;12(1):128. doi: 10.3390/cancers12010128 (PMC7017236; doi:10.3390/cancers12010128)
Supplement: Supplementary file 1 [file cancers-12-00128-s001.pdf]

STAT3 Inhibits CD103⁺ cDC1 Vaccine Efficacy in Murine Breast Cancer

Taylor T. Chrisikos, Yifan Zhou, Haiyan S. Li, Rachel L. Babcock, Xianxiu Wan, Bhakti Patel, Kathryn Newton, James J. Mancuso and Stephanie S. Watowich

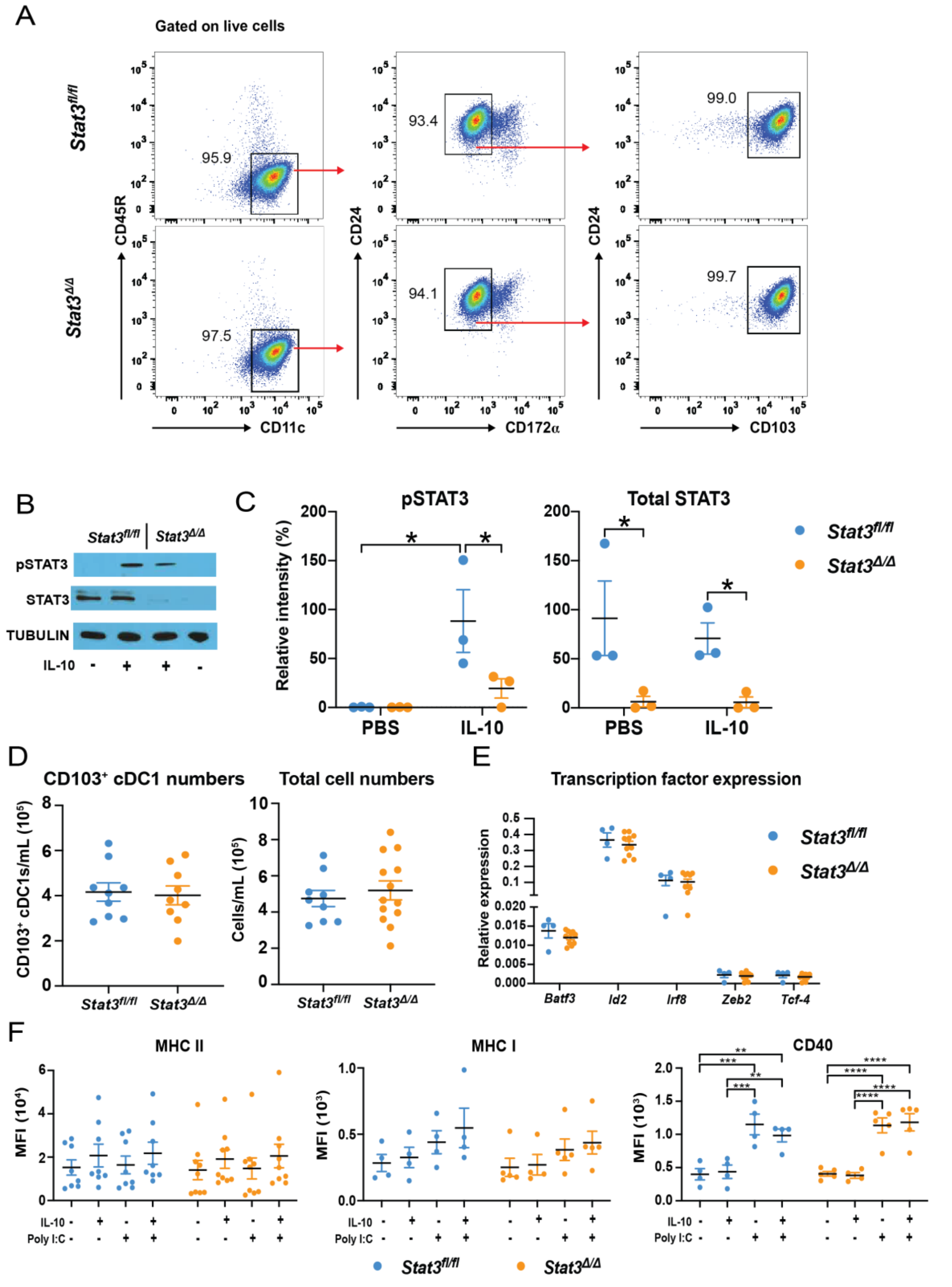

Figure S1. Characterization of *Stat3*-deficient CD103⁺ cDC1 cultures. (**A** – **F**) Analysis of in vitro-generated, FACS-purified *Stat3*-sufficent (*Stat3*$^{fl/fl}$) and *Stat3*-deficient (*Stat3*$^{\Delta/\Delta}$) CD103⁺ cDC1s. (**A**)

Representative flow cytometry plots demonstrating the gating scheme for FACS-purification of *Stat3*$^{fl/fl}$ and *Stat3*$^{\Delta/\Delta}$ CD103$^+$ cDC1s. Frequency of each parent population shown. (**B**) Representative immunoblots of Tyr705-phosphorylated STAT3 (pSTAT3), total STAT3, and tubulin from CD103$^+$ cDC1s following stimulation with (+) or without (-) IL-10 for 0.25 h. (**C**) Relative intensity of specific proteins normalized to tubulin, represented in (**B**), as indicated, determined using ImageJ software. Cumulative data from 3 independent experiments, $n = 3$. (**D**) CD103$^+$ cDC1 (left) and total culture (right) viable cell numbers after 17 d of culture, cumulative data from 5 independent experiments. CD103$^+$ cDC1 numbers, $n = 9$ (*Stat3*$^{fl/fl}$, *Stat3*$^{\Delta/\Delta}$); total culture density, $n = 9$ (*Stat3*$^{fl/fl}$), $n = 13$ (*Stat3*$^{\Delta/\Delta}$). (**E**) Relative mRNA expression of the indicated transcriptional regulators in CD103$^+$ cDC1s after 17 d of culture. Data are cumulative from 3 independent experiments. $n = 4$ (*Stat3*$^{fl/fl}$), $n = 10$ (*Stat3*$^{\Delta/\Delta}$). (**F**) MHC II, MHC I, and CD40 surface expression on FACS-purified CD103$^+$ cDC1s, 16 h following treatment with PBS, 10 ng/mL murine IL-10, 20 µg/mL poly I:C, or 10 ng/mL IL-10 and 20 µg/mL poly I:C, as indicated. MHC II, $n = 8$ (*Stat3*$^{fl/fl}$), $n = 9$ (*Stat3*$^{\Delta/\Delta}$) for all treatments, combined from 4 independent experiments. MHC I and CD40, $n = 4$ (*Stat3*$^{fl/fl}$), $n = 5$ (*Stat3*$^{\Delta/\Delta}$) for all treatments, combined from 3 independent experiments. (**C**-**F**) Data are shown as mean ± S.E.M. (**C**, **F**) Data analyzed by two-way ANOVA and Bonferroni's multiple comparison test, comparing all treatments within each genotype, and comparing the two genotypes within a given treatment, or (**D**) unpaired, two-tailed *t* test, or (**E**) two-way ANOVA and Bonferroni's multiple comparison test. Results were considered significant when $p < 0.05$. ** $p < 0.01$, *** $p < 0.001$, **** $p < 0.0001$.

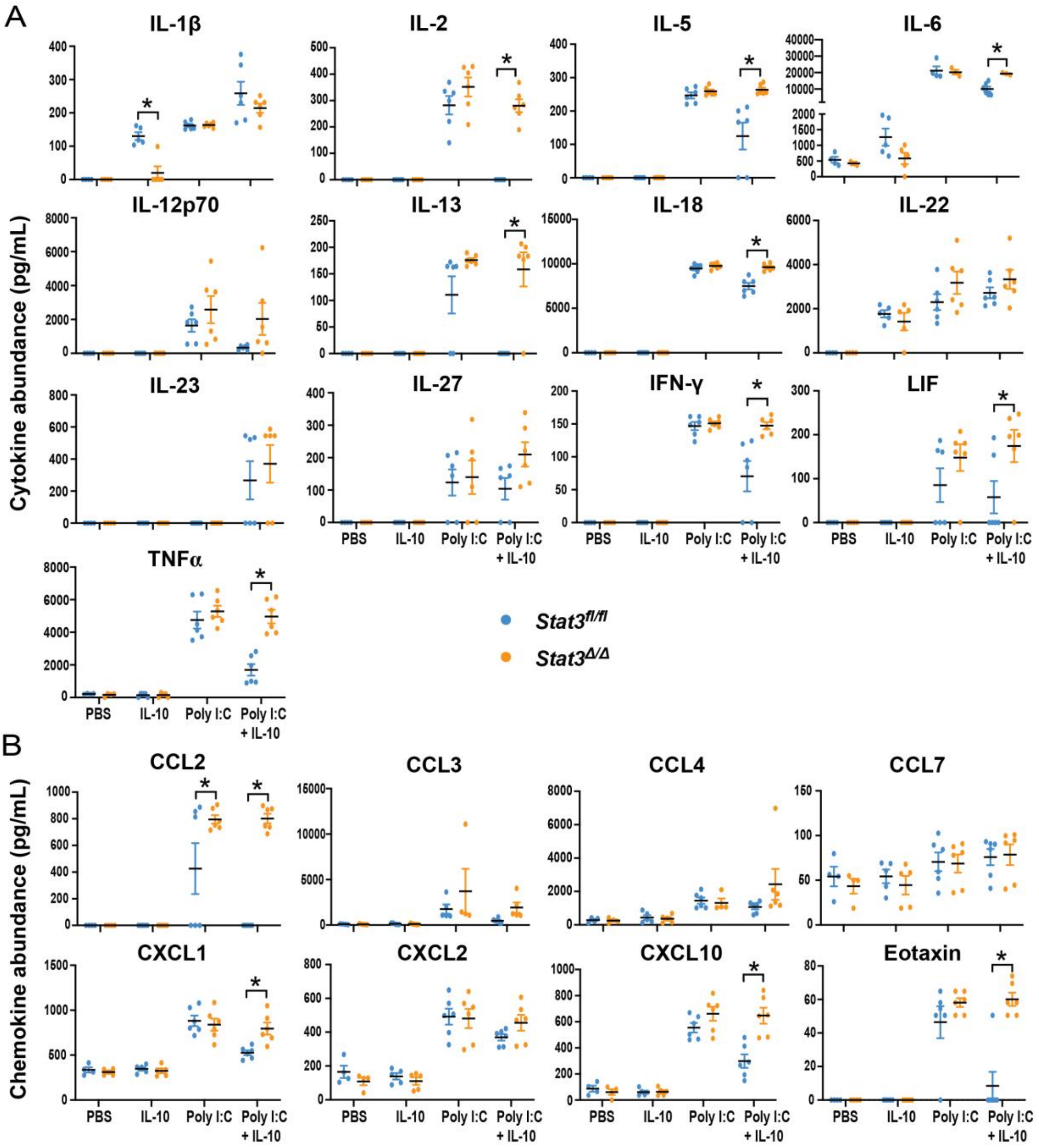

Figure S2. Cytokine and chemokine expression in *Stat3*-deficient CD103⁺ cDC1s. In vitro-generated, FACS-purified $Stat3^{fl/fl}$ or $Stat3^{\Delta/\Delta}$ CD103⁺ cDC1s were stimulated with or without 10 ng/mL murine IL-10, 20 µg/mL poly I:C, or 10 ng/mL IL-10 and 20 µg/mL poly I:C, as indicated. Culture supernatants were analyzed for cytokine (**A**) and chemokine (**B**) amounts using multiplexed assays. $n = 4$ (PBS), $n = 5$ (IL-10), $n = 6$ (pI:C), $n = 6$ (pI:C + IL-10), for both genotypes, combined from 4 independent experiments. Data are shown as mean ± S.E.M. Data analyzed by two-way ANOVA and Bonferroni's multiple comparison test. Results were considered significant when $p < 0.05$. * $p < 0.05$.

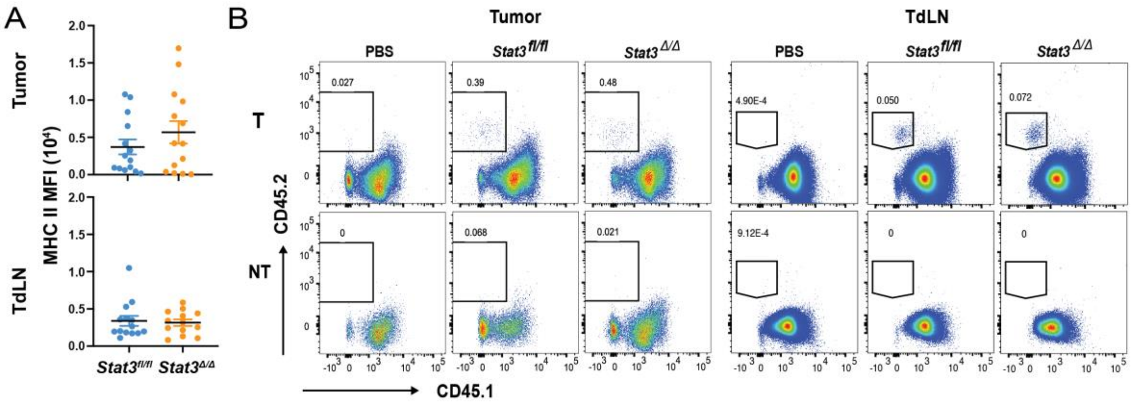

Figure S3. Maturation and migration of *Stat3*-deficient CD103$^+$ cDC1s after intratumoral injection. CD45.1$^+$ mice bearing bilateral PyMT-OVA tumors received i.t. injection of PBS, the CD45.2$^+$ $Stat3^{fl/fl}$ CD103$^+$ cDC1 vaccine, or the CD45.2$^+$ $Stat3^{\Delta/\Delta}$ CD103$^+$ cDC1 vaccine in right side tumors only, 7 d after tumor establishment. Vaccine-derived CD103$^+$ cDC1s were identified by analysis of CD45.2$^+$ cells 40 h after i.t. injection. (**A**) Surface MHC II expression on vaccine-derived CD45.2$^+$ CD103$^+$ cDC1s in tumors or TdLNs, as indicated. Data combined from 2 independent experiments. n = 14 ($Stat3^{fl/fl}$ tumor, TdLN; $Stat3^{\Delta/\Delta}$ tumor), n = 13 ($Stat3^{\Delta/\Delta}$ TdLN). (**B**) Representative flow cytometry plots analyzing vaccine-derived CD45.2$^+$ CD103$^+$ cDC1s in treated and non-treated tumors and TdLNs, as indicated. Data are representative of 2 independent experiments. n = 6 (PBS tumor, TdLN), n = 14 ($Stat3^{fl/fl}$ tumor, TdLN; $Stat3^{\Delta/\Delta}$ tumor), n = 13 ($Stat3^{\Delta/\Delta}$ TdLN). (**A**) Data shown are mean ± S.E.M. Data analyzed by unpaired, two-tailed t test. Results were considered significant when $p < 0.05$.

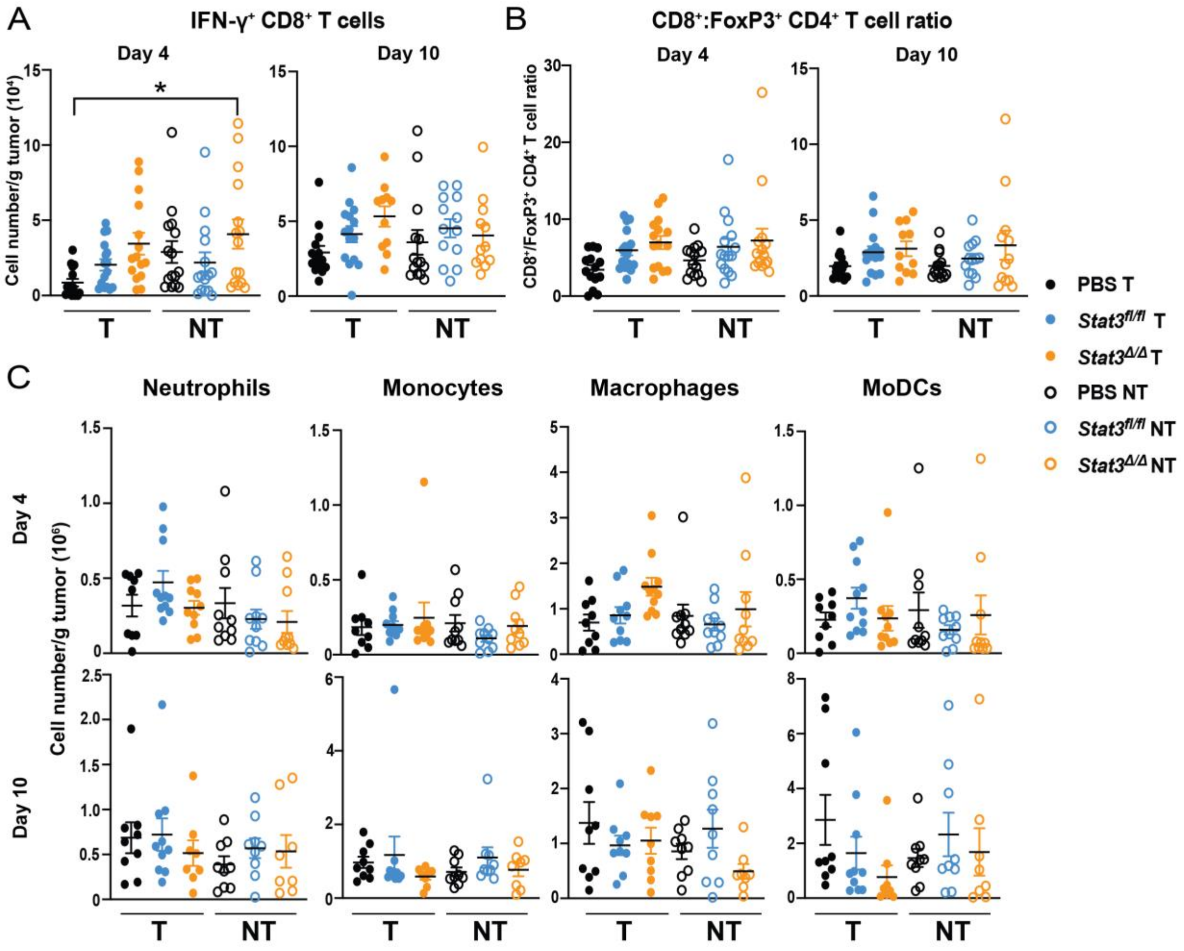

Figure S4. T cell and myeloid responses to Stat3-deficient CD103^{+} cDC1 vaccine. Immune subsets were analyzed in tumors 4 d and 10 d following i.t. injection of PBS, the *Stat3*$^{fl/fl}$ CD103^{+} cDC1 vaccine, or the *Stat3*$^{\Delta/\Delta}$ CD103^{+} cDC1 vaccine, in right side tumors of bilateral PyMT-OVA tumor-bearing mice. (**A**) Cumulative amounts of IFN-γ^{+} CD8^{+} T cells in tumors at the indicated time points. (**B**) Cumulative ratio of CD8^{+}:FoxP3^{+} CD4^{+} T cells. (**A**, **B**) Data from 3 independent experiments. Tumors, d 4, n = 15 (PBS T, NT; *Stat3*$^{fl/fl}$ NT; *Stat3*$^{\Delta/\Delta}$ T, NT), n = 16 (*Stat3*$^{fl/fl}$ T). TdLNs, d 4, n = 15 for all groups. Tumors, d 10, n = 14 (PBS T, NT), n = 15 (*Stat3*$^{fl/fl}$ T), n = 11 (*Stat3*$^{\Delta/\Delta}$ T), n = 13 (*Stat3*$^{fl/fl}$ NT), n = 12 (*Stat3*$^{\Delta/\Delta}$ NT). TdLNs, d 10, n = 13 (PBS T), n = 14 (PBS NT), n = 15 (*Stat3*$^{fl/fl}$ T, NT; *Stat3*$^{\Delta/\Delta}$ T, NT) (**C**) Cumulative amounts of neutrophils (CD11b^{+} Ly6G^{+}), monocytes (CD11b^{+} Ly6G^{-} Ly6C^{hi} F4/80^{lo}), macrophages (CD11b^{+} Ly6G^{-} Ly6C^{lo} F4/80^{hi} CD11c^{-} MHC II^{-}), and moDCs (CD11b^{+} Ly6G^{-} Ly6C^{lo} F4/80^{hi} CD11c^{+} MHC II^{hi}) in tumors at the indicated time points, from 2 independent experiments. For d 4, n = 9 (PBS T), n = 11 (*Stat3*$^{fl/fl}$ T), n = 10 (PBS NT; *Stat3*$^{fl/fl}$ NT; *Stat3*$^{\Delta/\Delta}$ T, NT). For d 10, n = 9 (PBS T, NT; *Stat3*$^{fl/fl}$ NT), n = 10 (*Stat3*$^{fl/fl}$ T), n = 8 (*Stat3*$^{\Delta/\Delta}$ T, NT). (**A**, **B**, and **C**) Data are shown as mean ± S.E.M. Data analyzed by one-way ANOVA and Tukey's multiple comparison test. Results were considered significant when $p < 0.05$. * $p < 0.05$.

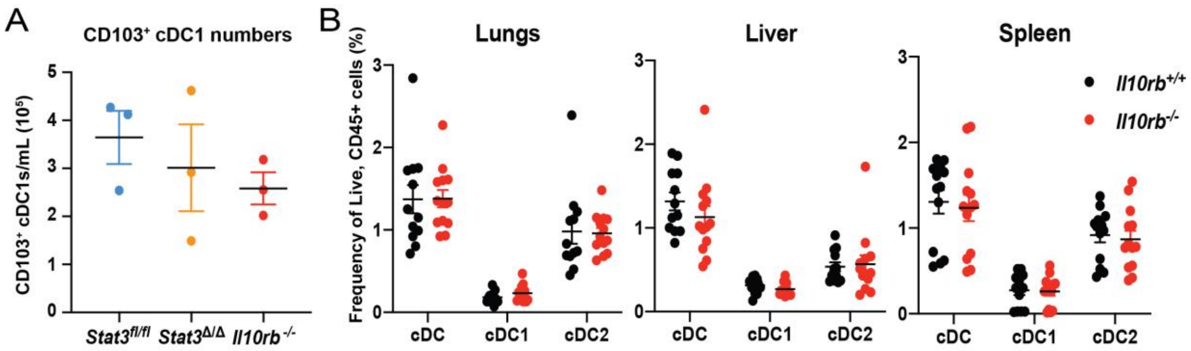

Figure S5. *Il10rb* is dispensable for CD103^{+} cDC1 development. (**A**) Cumulative CD103^{+} cDC1 viable cell numbers on d 17 of culture, from 3 independent experiments, n = 3 for all groups. (**B**) Cumulative frequency of cDCs (CD45^{+} CD3^{-} CD19^{-} F4/80^{-} CD11c^{+} MHC II^{hi}), cDC1s (CD45^{+} CD3^{-} CD19^{-} F4/80^{-} CD11c^{+} MHC II^{hi} XCR1^{+} CD172α^{-}), and cDC2s (CD45^{+} CD3^{-} CD19^{-} F4/80^{-} CD11c^{+} MHC II^{hi} XCR1^{-} CD172α^{+}), in the indicated organs from 2 independent experiments. n = 12 (*Il10rb*$^{+/+}$), n = 13 (*Il10rb*$^{-/-}$). (**A**,**B**) Data are shown as mean ± S.E.M. (**A**) Data analyzed by one-way ANOVA and Tukey's multiple comparison test or (**B**) by unpaired, two-tailed t test. Results were considered significant

Supplementary Methods

Immunoblotting

CD103^{+} cDC1 cell lysates were subjected to SDS-PAGE and immunoblotting with antibodies to detect Tyr705-phosphorylated STAT3 (pSTAT3) (Cell Signaling Technology, Danvers, Massachusetts, USA), total STAT3 (Santa Cruz Biotechnology, Dallas, Texas, USA), or tubulin (clone 12G10). Relative intensity of pSTAT3 and STAT3 signals versus tubulin signal was determined using ImageJ software.

RNA isolation and quantitative polymerase chain reaction (qPCR)

RNA extraction was performed using TRIzol™ (Invitrogen) and RNA reverse-transcription into cDNA was done using the iScript™ cDNA Synthesis Kit (Bio-Rad, Hercules, CA, USA), each in accordance with the manufacturer's instructions. To evaluate relative gene expression, qPCR was performed using SYBR Green (Millipore Sigma) on a CFX384 Touch™ Real Time PCR Detection System (Bio-Rad) with the following protocol: denaturation at 95°C for 10 seconds (s); annealing and extension at 60°C for 20 s. mRNA expression of target genes was normalized to ribosomal protein L13 (*Rpl13)* mRNA as an endogenous control. The following primers were used: *Rpl13* forward (F) 5′-GGCTGAAGCCTACCAGAAAG-3′, *Rpl13* Reverse (R) 5′-

TTCTCCTCCAGAGTGGCTGT-3′; *Batf3* F 5′-CAGACCAGAAGGCTGACAAG-3′, *Batf3* R 5′-CTGCGCAGCACAGAGTTCTC-3′; *Id2* F 5′-AAACAGCCTGTCGGACCAC-3′, *Id2* R 5′-CTGGGCACCAGTTCCTTGAG-3′; *Irf8* F 5′-GAGCCAGATCCTCCCTGACT-3′, *Irf8* R 5′-GGCATATCCGGTCACCAGT-3′; *Tcf-4* F 5′-AGACCAAGCTCCTGATTCTC-3′, *Tcf-4* R 5′-AGGCTCTGAGGACACCTTCT-3′; *Zeb2* F 5′-GGCAAGGCCTTCAAGTACAA-3′, *Zeb2* R 5′-AAGCGTTTCTTGCAGTTTGG-3′.

Immune cell isolation from lungs, liver, and spleen

Lungs and livers were removed and cut into small pieces (~2 mm) and incubated in RPMI 1640 medium (Thermo Fisher Scientific, Waltham, MA, USA) containing 1 mg/mL collagenase type IV (Millipore Sigma, Darmstadt, Germany) for 45 minutes (min) in a shaking incubator (Eppendorf New Brunswick Excella E25) at 37°C and 100 RPM. Digested lung and liver suspensions were passed through 100 μm mesh filters. Lung suspensions were subsequently washed with PBS containing 2 mM ethylenediaminetetraacetic acid (EDTA) and 2% FBS (FACS buffer) in preparation for antibody staining. Liver suspensions were enriched for immune cells using Percoll (GE Healthcare Life Sciences, Pittsburgh, Pennsylvania, USA) density gradient centrifugation; liver suspensions were resuspended in 37% Percoll, layered on top of 70% Percoll and centrifuged for 25 min at 1200 x g at room temperature, without brake. Cells at the interface of the two Percoll layers were harvested and subsequently washed with FACS buffer in preparation for antibody staining. Spleens were removed and passed through 100 μm mesh filters and washed with RPMI. Spleen suspensions were then exposed to RBC lysis buffer (Tonbo Biosciences, San Diego, California, USA) for 5 min at room temperature and subsequently washed with FACS buffer in preparation for antibody staining.

cDC abundance determination by antibody staining and flow cytometry

Single cell suspensions were stained for surface markers and analyzed as described in the Methods section in the main text. The following reagents were used: BV-421-conjugated XCR1 (ZET) antibody; redFluor™ 710-conjugated CD45.2 (104) antibody; Percp-Cy5.5-conjugated CD3 (17A2), F4/80 (BM8), and CD19 (1D3) antibodies; APC-Cy7-conjugated CD11c (N418) antibody; PE-Cy7-conjugated anti-MHC II (M5/114.15.2) antibody; and APC-conjugated CD172α (P84) antibody.

© 2020 by the authors. Licensee MDPI, Basel, Switzerland. This article is an open access article distributed under the terms and conditions of the Creative Commons Attribution (CC BY) license (http://creativecommons.org/licenses/by/4.0/).
